# Identification of specific genes as molecular markers for rapid and accurate detection of oil-tea *Camellia* anthracnose pathogen *Colletotrichum fructicola* in China

**DOI:** 10.3389/fmicb.2024.1442922

**Published:** 2024-08-26

**Authors:** Lingxue Cao, Kailin Shi, Yongyi Liu, Xiaonan Xie, Xizhe Sun, Wentong Dong, Congya Wang, Lisong Ma

**Affiliations:** ^1^Laboratory of Cultivation and Protection for Non-Wood Forest Trees, Central South University of Forestry and Technology, Changsha, China; ^2^Key Laboratory of Forest Bio-resources and Integrated Pest Management for Higher Education in Hunan Province, Central South University of Forestry and Technology, Changsha, China; ^3^State Key Laboratory of North China Crop Improvement and Regulation, College of Horticulture, Hebei Agricultural University, Baoding, China; ^4^Hunan Tianhua Tea-oil Technology, Changsha, China

**Keywords:** *Colletotrichum fructicola*, oil-tea *Camellia*, molecular markers, pathogen detection, end-point PCR, qPCR

## Abstract

**Introduction:**

*Camellia* anthracnose is caused by multiple *Colletotrichum* species, resulting in severe yield losses of oil-tea *Camellia*. *Colletotrichum fructicola* is one of the major anthracnose pathogens of oil-tea *Camellia* worldwide. However, developing unique molecular markers for the rapid and accurate detection of *Colletotrichum fructicola* from diverse *Colletotrichum* species, as well as early monitoring and effective control of the disease, remains largely unexplored.

**Methods:**

*C. fructicola*-specific genes were obtained using a BLAST search of the sequences of predicted genes in *C. fructicola* against the genome sequences of *Colletotrichum* fungal pathogens. In this study, *Colletotrichum fructicola*-specific molecular markers were developed for rapid and accurate detection of *C. fructicola* among *Camellia* anthracnose causing fungal pathogens.

**Results:**

Using genomic DNA-based end-point PCR and qPCR, three *C. fructicola*-specific genes with the ability to distinguish *C. fructicola* from other oil-tea *Camellia* anthracnose-related *Colletotrichum* species, including *Colletotrichum camelliae, Colletotrichum gloeosporioides*, and *Colletotrichum siamense*, and oil-tea *Camellia* fungal pathogens belonging to the genus *Neopestalotiopsis, Pestalotiopsis*, and *Alternaria*, were validated as molecular markers. In addition, these three molecular markers were highly sensitive to detecting *C. fructicola* using DNA extracted from the inoculated leaves of oil-tea *Camellia*.

**Discussion:**

These findings enable us to rapidly and uniquely detect the *Camellia* anthracnose disease caused by *Colletotrichum fructicola*, which will equip farmers with an effective tool for monitoring *Camellia* anthracnose disease in the field and taking timely control measurements in advance.

## 1 Introduction

Anthracnose caused by *Colletotrichum* species is a devastating disease on oil-tea *Camellia oleifera* (Wang et al., 2020; Chen et al., 2022). The *Colletotrichum* pathogens, including *Colletotrichum camelliae, Colletotrichum fructicola, Colletotrichum siamense, Colletotrichum aenigma* and *Colletotrichum gloeosporioides*, have been reported to infect the buds, fruits and leaves of oil-tea *Camellia oleifera* in southern China (Jin et al., 2009; Chen et al., 2022, 2023). With the expanded cultivation of oil-tea *Camellia* in China, epidemics of anthracnose disease increasingly occur, resulting in reduced annual production of oil tea ranging from 10 to 30% (Qing et al., 2009). *C. fructicola* is the most predominantly identified species in diseased leaves of oil tea, followed by *C. camelliae* (Li et al., 2016). *C. fructicola* that was first isolated from diseased *Coffea arabica* in northern Thailand is a dominant pathogen of oil-tea anthracnose, belonging to the *C. gloeosporioides* complex (Prihastuti et al., 2009). As a member of *Colletotrichum* genus that is ranked among the top 10 plant pathogenic fungi (Dean et al., 2012), *C. fructicola* has the ability to cause anthracnose, bitter rot, and leaf spot diseases on a wide range of woody or herbaceous plants worldwide (Cannon et al., 2012; Fu et al., 2019; Li et al., 2021, 2022; Martin et al., 2021; Evallo et al., 2022; Zhao et al., 2022). The Chinese native commercial shrub oil-tea *Camellia* is threatened by *C. fructicola* because the pathogen can produce a large number of conidia spores, which are primary initial and secondary infection resources and can be easily disseminated to neighboring healthy blossoms, young fruit or leaves by rain splash, wind-driven rain and human conducted cultivation activities (De Silva et al., 2017; Chen et al., 2021).

Distinguishing specific pathogen species can facilitate the selection of an adequate disease management strategy (Cai et al., 2011). Quick and accurate detection and differentiation of different *Colletotrichum* species is essential in monitoring the oil-tea *Camellia* anthracnose epidemic to control the spread of the disease. Diagnosing plant diseases routinely depends on symptom observation and fulfillment of Koch's postulates in combination with morphological and molecular characterization (Damm et al., 2010; Liu et al., 2016). Currently, lab-based molecular identification and quantification of fungal pathogens using qPCR and multilocus phylogenetic analyses based on DNA sequences of the ribosomal internal transcribed spacer regions, calmodulin, β-tubulin, actin, chitin synthase-encoding genes and glyceraldehyde-3-phosphate dehydrogenase facilitate the differentiation of individual *Colletotrichum* species (Chen et al., 2022). For instance, multilocus phylogenetic analysis based on multiple gene sequences has been used as a supplement to morphological analysis to reliably and accurately identify *C. fructicola* within the *C. gloeosporioides* complex, including morphologically and physiologically identical 22 species plus one subspecies (Weir et al., 2012; Giblin et al., 2018; Grammen et al., 2019; Guarnaccia et al., 2021). In addition, based on multilocus phylogenetic analysis in combination with morphological characterization, 106 *Colletotrichum* isolates obtained from leaves of *Camellia sinensis* with anthracnose symptoms in the tea-growing regions of China have been identified to belong to 11 *Colletotrichum* species (Wang et al., 2016). The identification of 232 isolates collected from diseased leaves and fruits of oil-tea *Ca. oleifera* based on morphology and multilocus phylogenetic analyses found that they belong to five species, including *C. camelliae, C. fructicola, C. siamense, C. aenigma*, and *C. gloeosporioides* (Wang et al., 2020). Although multi-gene phylogenetic analysis coupled with morphological characterization has been successfully used to differentiate *C. fructicola* from multiple *Colletotrichum* species, the identification process is time-consuming, labor-intensive, and cost-effective. Therefore, the development of a single molecular marker for fast and accurate detection of *C. fructicola* from complex *Colletotrichum* species is required.

In this study, BLAST search of the predicted coding gene sequences of *C. fructicola* against the genomes of three anthracnose *Colletotrichum* species, including *Colletotrichum camelliae, Colletotrichum gloeosporioides*, and *Colletotrichum siamense*, was conducted to identify *C. fructicola*-unique coding sequences. Specific primers based on *C. fructicola*-unique coding sequences were developed and examined for the detection of *C. fructicola* using DNA-based end-point PCR and qPCR. Finally, three primer sets with high accuracy are obtained to detect and differentiate *C. fructicola* from other oil-tea *Camellia* anthracnose-related fungal species using end-point PCR and qPCR with DNA extracted from the diseased plant tissue.

## 2 Materials and methods

### 2.1 Fungal isolates and cultivation

The oil-tea *Camellia* anthracnose-related fungal species were originally isolated from diseased leaves and stored at the Key Laboratory of National Forestry and Grassland Administration for Control of Diseases and Pests of South Plantation, Central South University of Forestry and Technology, Changsha, China (Li et al., 2016). *Colletotrichum* spp. [*Colletotrichum camelliae, Colletotrichum gloeosporioides, Colletotrichum siamense* and *Colletotrichum fructicola* (CFLH16)], together with three other fungal strains belonging to the genus *Neopestalotiopsis, Pestalotiopsis* and *Alternaria*, were recovered from −80°C glycerol stocks and cultured in potato dextrose broth (PDB) medium shaking at 150 rpm and temperature of 28°C for 5 days to produce conidia.

### 2.2 *Colletotrichum frucitcola* conidia inoculation

Conidia preserved at −80°C were first recovered on solid potato dextrose agar (PDA) plate for 5 days at 28°C, and then the hyphae were transferred to PDB medium shaking at 150 rpm and temperature of 28°C for another 5 days. *C. fructicola* conidia were harvested by filtering a 5-day-old fungal liquid culture through three layers of Miracloth (Merck Millipore, Darmstadt, Germany). After washing with sterile water three times, the conidia were resuspended to 10^5^ spores mL^−1^ in sterile water with 0.01% (v/v) Tween-20 for oil-tea *Camellia* inoculation. Surface sterilized *Camellia* leaves were placed in a Petri dish with the abaxial side up, and a volume of 1 mL of this spore suspension was sprayed using a handheld sprayer on the plant leaves per Petri dish. The treated materials were then cultivated in Petri dishes to keep humid for up to 5 days at 28°C before being harvested for total genomic DNA extraction.

### 2.3 DNA extraction from fungal conidia and oil-tea *Camellia* leaves

Fungal conidia were individually isolated by filtrate 5-day-old fungal PDB culture through three layers of Miracloth, and were washed with sterile water three times. Healthy *Ca. oleifera* leaves were collected from the orchard in the campus of Central South University of Forestry and Technology, Changsha, China. The conidia samples and *Ca. oleifera* leaves were frozen in liquid nitrogen, and these samples were ground to fine powder by Mixer Mill MM400 (Retsch, Haan, Germany). The total genomic DNA was isolated using CTAB method. For 500 mg fungal or botanic material, or inoculated oil-tea *Camellia* leaves at 5 days with *C. fructicola* conidia 1 mL CTAB extraction buffer supplemented with 10 μL β-mercaptoethanol was added and then vortexed to mix the sample powder with CTAB thoroughly before incubation at 65°C for 1 h. These CTAB mixtures were then cooled at room temperature for 1 h and gently mixed with an equal volume of chloroform. The extraction buffers were centrifuged at 10,000 g for 10 min to isolate the supernatant individually. The supernatant was transferred to a new centrifuge tube with an equal volume of isopropanol to precipitate total genomic DNA by gently mixing the supernatant with isopropanol. The DNA was collected as a pellet with centrifugation at 10,000 g for 10 min, and the DNA pellet was washed twice with ice-cold 70% (v/v) ethanol before dissolving in 100 μL distilled water. The total genomic DNA was quantified using the Eppendorf BioPhotometer Plus (Eppendorf, Hamburg, Germany).

### 2.4 Sequence analysis and primer design

Genome sequences of *Colletotrichum camelliae, Colletotrichum gloeosporioides, Colletotrichum siamense*, and *Colletotrichum fructicola* were downloaded from the NCBI genome database (https://www.ncbi.nlm.nih.gov/genome/). All coding sequences of *C. fructicola* were aligned to the genomes of *Colletotrichum camelliae, Colletotrichum gloeosporioides*, and *Colletotrichum siamense*, respectively, using BLASTN with an e-value cutoff value of 1e^−5^. The coding sequence without any BLAST hits against other *Colletotrichum* species was selected, and the genes without intron were taken as the candidate markers for *Colletotrichum fructicola* identification. Primers targeting selected *C. fructicola* unique genes were designed using Primer5 software (Supplementary Table S1).

### 2.5 End-point PCR amplification

Each PCR reaction was performed in a 20 μL reaction mixture comprised of 1 μL DNA template, 1 μL of 10 μ mol/L forward and reverse primer (10 μM of each primer), 10 μL 2 × Es Taq MasterMix (Cwbio, Taizhou, Jiangsu, China), and 7 μL distilled water. The PCR program used to produce proper amplicons was: initial denaturation at 94°C for 2 min; 35 cycles of 94°C denaturations for 30 s, annealing at 69°C for 30 s, extension at 72°C for 10 s; followed by a final extension at 72°C for 2 min on a Mastercycler nexus (Eppendorf, Hamburg, Germany). The PCR products were examined by 2% agarose gel electrophoresis. This experiment was repeated three times, and the same results were obtained.

### 2.6 qPCR assay

qPCR was performed using a 7500 Real-time PCR System (Applied Biosystems, Foster City, CS, USA) with T5 Fast qPCR Mix (SYBR Green I) (Tsingke Biotechnology, Beijing, China), in a final volume of 20 μL containing 0.1 μM of each primer from the respective primer set, 1 μL genomic DNA. The real-time PCR amplification program consisted of an initial denaturation step at 95°C for 4 min, followed by 40 cycles of denaturation for 15 s at 95°C, annealing for 30 s at 69°C and extension for 30 s at 72°C. Melt curve analysis was carried out following amplification by continuously heating from 60 to 95°C, with the acquisition of fluorescence at 0.3°C intervals and a 15 s hold at each increment.

## 3 Results

### 3.1 Identification of *C. fructicola*-specific genes for primer design

**Figure 1:**
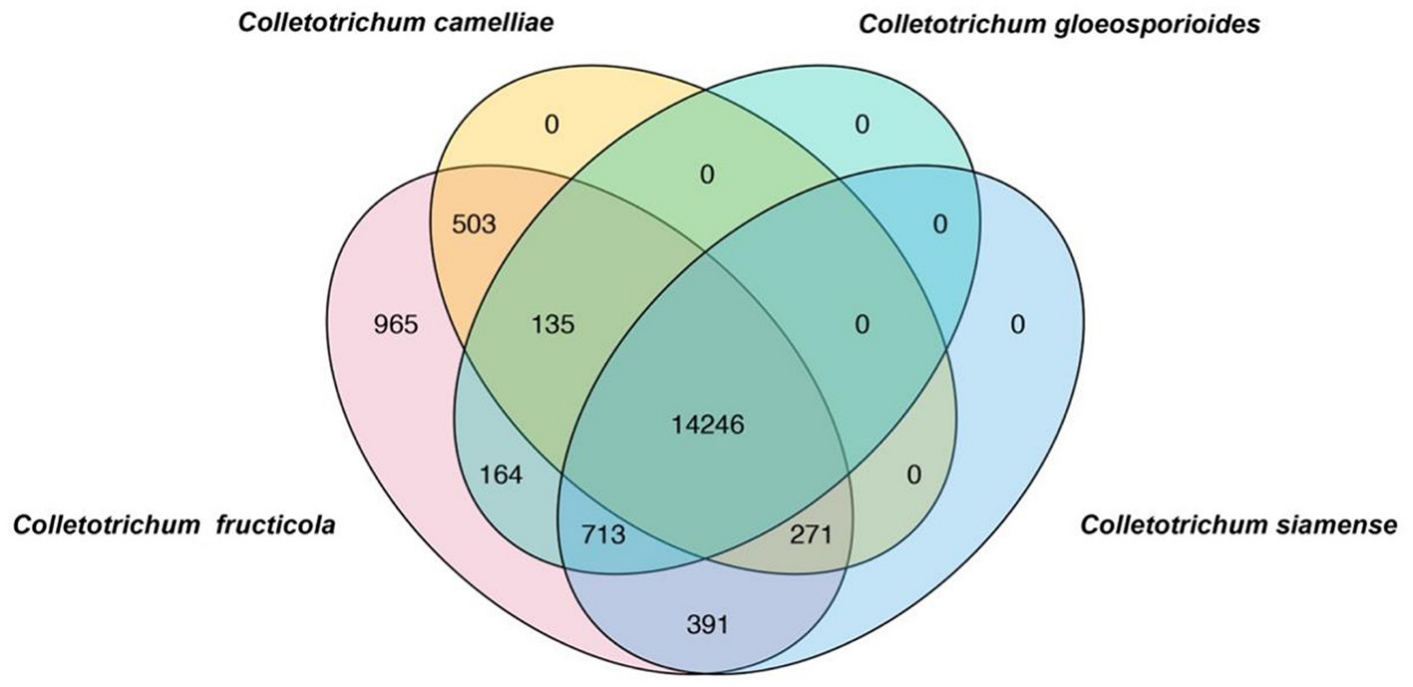
Venn diagrams showing the unique genes in different *Colletotrichum* species. BLAST search was conducted using all coding sequences of *C. fructicola* against the genome sequences of *Colletotrichum camelliae, Colletotrichum gloeosporioides*, and *Colletotrichum siamense*. *C. fructicola-*specific genes are indicated by pink.

To rapidly and simply differentiate *C. fructicola* from other *Colletotrichum* species, we conducted a BLAST search of coding sequences of *C. fructicola* against the genome sequences of *C. camelliae, C. gloeosporioides, C. siamense*, respectively. Nine hundred and sixty-five *C. fructicola*-specific gene-coding sequences were obtained (Figure 1). Based on the predicted gene function and annotation from NCBI, 115 *C. fructicola*-specific genes encoding proteins participating in various cellular processes and interactions with the environment that are essential for fungal survival and reproduction were selected from 965 *C. fructicola* specific genes (Figure 1). To design *C. fructicola* specific primers that can be used for genomic DNA-based PCR detection, 22 genes without intron were selected (Supplementary Figure S1). qPCR primer pairs were designed to target these *C. fructicola*-specific gene regions (Supplementary Table S1).

### 3.2 Validation of *C. fructicola* primers specificity by end-point PCR

**Figure 2:**
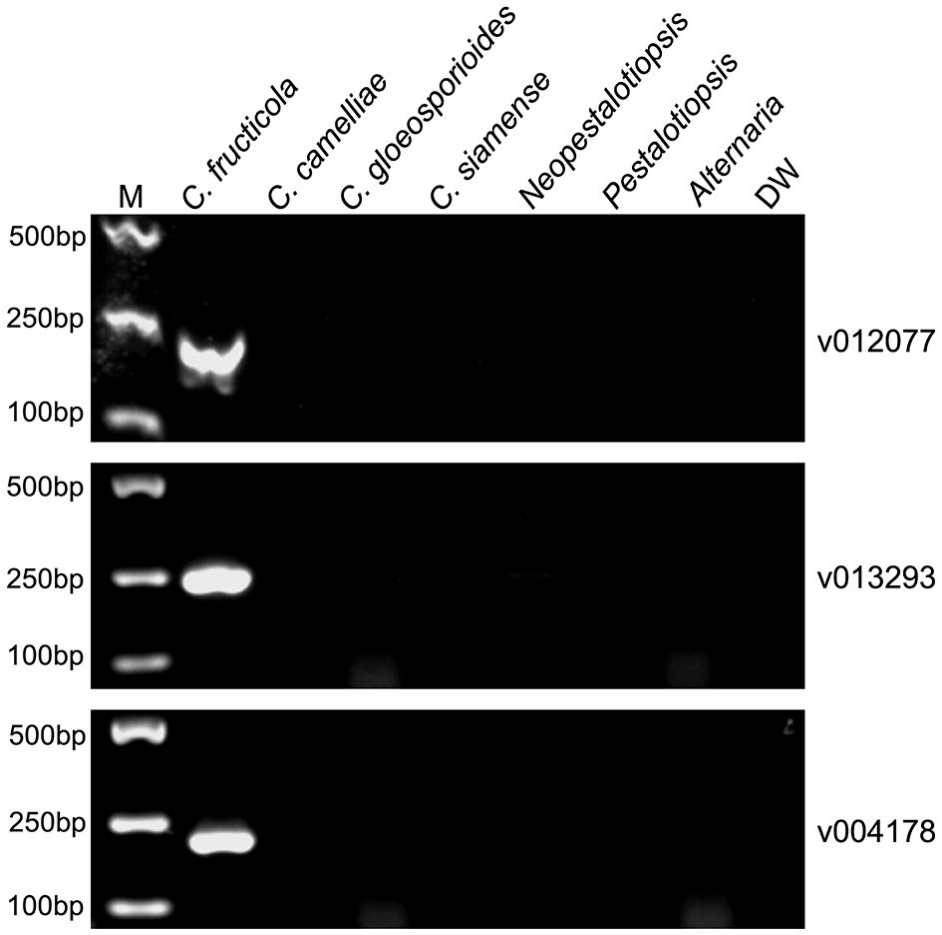
PCR amplification using three *C. fructicola*-specific primer sets to determine their presence in different *Camellia* anthracnose-related fungal pathogens. End-point PCR with primer pairs targeting different *C. fructicola* genes v012077, v013293, and v004178 was performed, respectively. The genomic DNAs of each oil-tea *Camellia* anthracnose-related fungal pathogens were used as templates. M: DL2000 Marker. DW: distilled water.

To evaluate the specificity of the designed *C. fructicola* primer sets, the genomic DNA extracted from four oil-tea *Camellia* pathogenic *Colletotrichum* spp. and the other three fungal genera isolated from diseased leaves of oil-tea *Camellia* anthracnose were used as the template for end-point PCR amplification with different primer sets, respectively. Among the 22 *C. fructicola*-specific primer pairs, end-point PCR amplification with three primer sets targeting v012077 (putative AC transposase), v013293 (nonribosomal peptide synthetase dtxS1), and v004178 (acyltransferase easC) produced specific bands with the expected sizes of 215, 243, and 208 bp, respectively, while no amplicons were generated from *C. camelliae, C. gloeosporioides, C. siamense, Neopestalotiopsis, Pestalotiopsis* and *Alternaria* (Figure 2). In addition, we optimized the annealing temperature of each primer pair and found that end-point PCR with the annealing temperature at 69°C produced the single amplicon of the target sequences (Figure 2). Taken together, these findings indicated that primer pairs v012077F/R, v013293F/R, and v004178F/R are specific for *C. fructicola* identification from the examined oil-tea *Camellia* anthracnose fungal pathogens.

### 3.3 Determining the specificity and efficiency of *C. fructicola*-specific primers using qPCR assay

**Figure 3:**
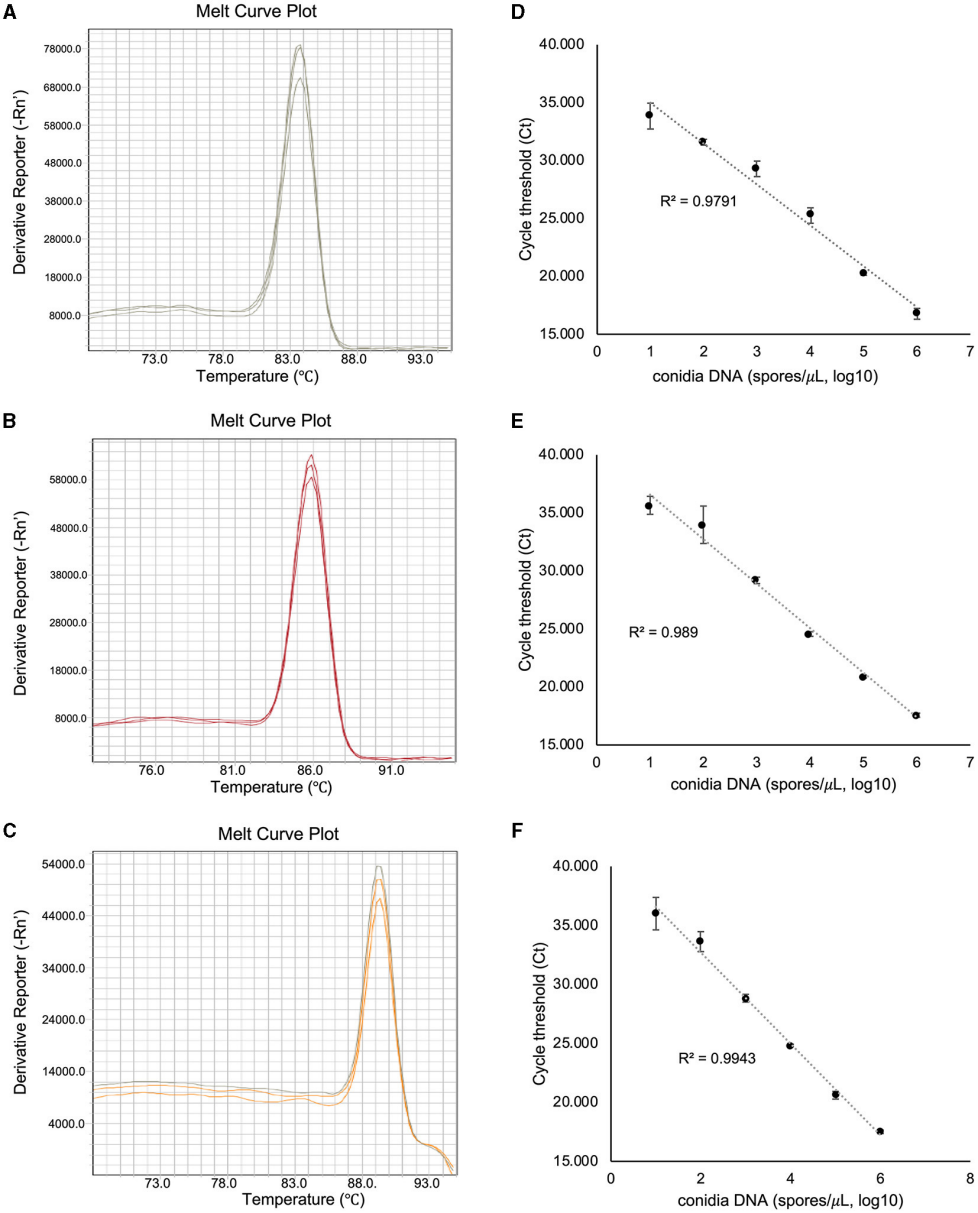
Evaluation of the amplification specificity and efficiency of *C. fructicola-*specific primers by qPCR. Melting curve analysis showing a single peak in each qPCR reaction with non-specific products was detected in *C. fructicola* v012077 **(A)**, v013293 **(B)**, and v004178 **(C)**, DNA extracted from *C. fructicola* conidia was used as the template for amplification. The primer efficiencies (*R*^2^) of *C. fructicola* v012077 **(D)**, v013293 **(E)**, and v004178 **(F)** primers were analyzed with a serial dilution of genomic DNA extracted from fungal conidia as templates. Error bars are standard deviations.

To assess the amplification specificity of v012077, v013293, and v004178 primers using qPCR assay, the melting curves were generated with the genomic DNA extracted from *C. fructicola* conidia. As shown in Figure 3A, the tested primer sets generated specific amplicons in qPCR assay, as evidenced by the appearance of a single peak in the melting curve in each sample. The amplification product of v012077 was specific, with a single peak around 83.7°C in the melting curve (Figure 3A). A single peak around 85.8°C in the melting curve of v013293 amplicons was observed in each sample, representing the specific amplification of this fragment (Figure 3B). The melting curve of v004178 products showed a single peak at 89.3°C (Figure 3C). These results suggested that the designed primer pairs targeting *C. fructicola* genes v012077, v013293, and v004178 can amplify specific PCR products using DNA-based qPCR assay. In addition, we performed qPCR assay using a set of 10-fold serially diluted *C. fructicola* genomic DNA to validate the primer efficiency. The amplification with v012077, v013293, and v004178 primer pairs produced a linear relationship between all diluted DNA templates and Ct values with a high correlation coefficient (*R*^2^ = 0.979–0.994) (Figures 3D–F). The high *R*^2^ value indicated that the Ct values are reliable predictors of the number of copies of the target gene detected in the qPCR assay. Overall, these results indicated that three *C. fructicola*-specific primer pairs were suitable for the sensitive detection of the target genes using genomic DNA-based qPCR.

### 3.4 Validating the sensitivity of *C. fructicola*-specific primers

**Figure 4:**
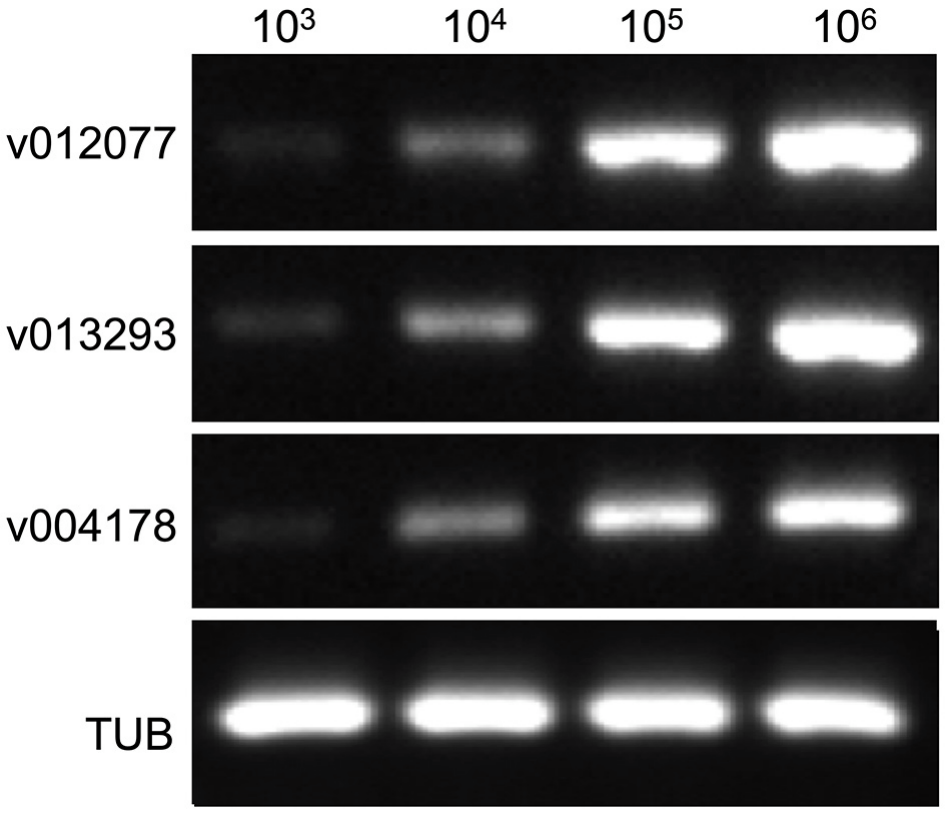
Determining the detection sensitivity of *C. fructicola-*specific primers via end-point PCR using *C. fructicola* and *Ca. oleifera* genomic DNA mixture. End-point PCR amplifications were performed with serial diluted *C. fructicola* conidial total DNA samples which were supplemented with oil-tea *Camellia* leaf genomic DNA. Oil-tea *Camellia* TUB was used as the plant inner control to indicate the plant genomic DNA in each reaction. M: DL2000 Marker. v012077, v013293, and v004178.

To determine the sensitivity of *C. fructicola* v012077, v013293, and v004178 primer pairs for the direct detection of *C. fructicola*, end-point PCR and qPCR were performed with a set of 10-fold serially diluted *C. fructicola* genomic DNA that mixed with *Ca. oleifera* DNA as templates. The oil-tea *Camellia* TUB primers were included as a positive control to show the presence of plant genomic DNA (Cao et al., 2023), and a single band of *TUB* amplicon was produced in each sample (Figure 4). The unique PCR products with the expected sizes of 215, 243, and 208 bp were observed in amplicons using the template DNA extracted from 10^4^ to 10^6^ spores, respectively. In addition, the sensitivity of the qPCR assay to detect *C. fructicola* was evaluated. Table 1 showed that the sensitivity of qPCR reached 1 spore (0.063 pg DNA of *C. fructicola*) for the v012077 primer set and 10 spores (0.63 pg DNA) for v013293 and v004178 primer pairs (Ct value < 35). These results indicated that three *C. fructicola*-specific primer pairs can be used to steadily detect *C. fructicola* by end-point PCR and qPCR without effects by *Camellia* DNA.

**Table 1:** Detection sensitivity of *C. fructicola*-specific primers using DNA extracted from a spore suspension with concentration ranging from 1 to 10^4^ spores/μL by qPCR.

**Target name**	**Conidia concentration (spores/μL DNA sample)**	**Ct values ±SD**
V012077	1	33.43 ± 0.42
V012077	10	32.34 ± 0.43
V012077	100	32.03 ± 0.60
V012077	1,000	28.84 ± 0.26
V012077	10,000	25.36 ± 0.28
V013293	1	35.36 ± 0.68
V013293	10	34.84 ± 0.05
V013293	100	32.70 ± 1.49
V013293	1,000	28.16 ± 0.37
V013293	10,000	23.64 ± 0.27
V004178	1	36.11 ± 1.13
V004178	10	33.83 ± 0.67
V004178	100	32.46 ± 0.88
V004178	1,000	27.34 ± 0.47
V004178	10,000	25.33 ± 0.36

### 3.5 Detection of *C. fructicola* in inoculated oil-tea *Camellia* leaves using qPCR

To validate the practical application of v012077, v013293, and v004178 primer pairs in directly detecting *C. fructicola* in *Ca. oleifera* leaves, the lower surface of detached *Ca. oleifera* leaves was inoculated with conidia (10^5^ spores/mL conidial suspension) by spraying. Leaf samples were harvested 5 days post inoculation for DNA extraction, and qPCR was then conducted to detect *C. fructicola*. As shown in Table 2, qPCR analysis using v012077, v013293, and v004178 primer sets can detect *C. fructicola* with Ct values 31.56, 34.04, and 33.79, respectively. The primer set of v012077 produced the lowest Ct value, indicating that the v012077 primer set is more sensitive than the other two primer sets. These results demonstrated the three *C. fructicola*-specific molecular markers based on qPCR technique can be used for direct detection of *C. fructicola* in inoculated oil-tea *Camellia* leaves, which can be used for practical surveillance of this pathogen.

**Table 2:** Detection of *C. fructicola* in inoculated oil-tea *Camellia* leaves by qPCR.

**Target name**	**Ct values ±SD**
V012077	31.56 ± 0.58
V013293	34.04 ± 0.83
V004178	33.79 ± 1.00

## 4 Discussion

*C. fructicola* is a versatile pathogen of the *C. gloeosporioides* complex, of which over 100 plant species have been reported as its hosts and the host list is continuing to grow (Bragard et al., 2021). As the predominant causal agent of oil-tea *Camellia* anthracnose, *C. fructicola* poses a significant threat to the oil-tea industry. Therefore, accurate detection and specific identification of *C. fructicola* are urgently required to safeguard oil-tea *Camellia* cultivation. The goal of this study was to develop molecular markers suitable for specific detection and identification of *C. fructicola* from oil-tea *Camellia*. In this work, we developed three *C. fructicola*-specific molecular markers to reliably detect *C. fructicola* in inoculated oil-tea *Camellia* leaves through DNA-based end-point PCR and qPCR approaches. We also determined the specificity of these three molecular markers in distinguishing *C. fructicola* from other oil-tea *Camellia* anthracnose-isolated *Colletotrichum* species, as well as from oil-tea *Camellia-*related fungal pathogenic genus *Neopestalotiopsis, Pestalotiopsis*, and *Alternaria*. This study provided a rapid and straightforward method to reliably distinguish *C. fructicola* from *C. gloeosporioides* complex, as well as from other oil-tea *Camellia* anthracnose-related fungal species in China.

*Colletotrichum* pathogens isolated from the leaves of *Ca. oleifera* anthracnose belong to *Colletotrichum gloeosporioides* complex (CGSC), and they are morphologically and physiologically identical or similar (Cannon et al., 2012; Weir et al., 2012). Therefore, the predominant oil-tea *Camellia* anthracnose pathogen *C. fructicola* cannot be reliably identified based on morphological characteristics. Since these *Ca. oleifera* pathogenic *Colletotrichum* species are genetically related, multilocus gene sequencing analysis is compulsory for *C. fructicola* identification (Li et al., 2016; Giblin et al., 2018; Grammen et al., 2019; Guarnaccia et al., 2021). Molecular markers for *C. fructicola* quick detection and identification with a single PCR procedure are urgently required. We developed three *C. fructicola-*specific molecular markers (v012077, v013293, and v004178) that are capable of distinguishing *C. fructicola* from other examined *Colletotrichum* species with genomic DNA-based end-point PCR and qPCR assays (Figures 2, 3). Previous studies showed that PCR primers targeting ribosomal internal transcribed spacer (ITS), calmodulin (CAL), β-tubulin (TUB2), actin, chitin synthase-encoding genes and glyceraldehyde-3-phosphate dehydrogenase (GAPDH) have been developed (Chen et al., 2022). The ribosomal internal transcribed spacer regions have been extensively used to design *Colletotrichum* species primers, such as *C. acutatum* detection with ribosomal internal transcribed spacer regions based primers from strawberry leaves and *C. abscissam*/*C. gloeosporioides* from Citrus leaves (Debode et al., 2009; Pereira et al., 2019). Besides these commonly used *Colletotrichum* identification regions, a cutinase gene-derived primer pair was used for indiscriminately distinguishing *C. gloeosporioides* complex, including *C. fructicola, C. gloeosporioides, C. aenigma* and *C. siamense*, from strawberry anthracnose (Yang et al., 2022). However, the high sequence similarity between these phylogenetic markers among species of *C. gloeosporioides* complex hindered the detection accuracy of these molecular markers and the development of *C. fructicola-*specific molecular markers. Reclassification of *C. fructicola* into *C. chrysophilum* has been reported by adding additional loci sequences to construct phylogenic tree. For example, *C. fructicola* isolated from diseased peach in the United States has been re-identified as *C. chrysophilum* by using eight loci analyses, including Actin (ACT), APN2 (DNA lyase gene, adjacent to MAT1-2-1 gene), ApMAT (the intergenic region between APN2 and partial sequence of the mating-type gene MAT1-2-1), CAL, GAPDH, GS (glutamine synthetase), ITS, and TUB2 (Khodadadi et al., 2020). Astolfi et al. (2022) reported that *C. fructicola* originally identified from glomerella leaf spot on apple in Southern Brazil and Uruguay was reclassified as *C. chrysophilum* by using APN2, ApMAT, CAL, and GS sequences in addition to original GAPDH, GD, and TUB2 sequences (Astolfi et al., 2022). These recent findings suggest that increasing the loci for the classification of *Colletotrichum* species is essential although the majority of identification and classification of *Colletotrichum* species causing anthracnose worldwide depends on the number of loci ranging from 3 to 5 (De Aguiar Carraro et al., 2022; Marins et al., 2022; Vieira et al., 2022). The *C. fructicola* isolate used in this study was originally isolated from the leaves of tea-oil *Camellia* anthracnose in China using four widely used loci sequences, including ITS, CL, GS, and GD (Li et al., 2016). *C. chrysophilum* has been increasingly reported to be responsible for anthracnose in America and Europe, while no studies reported that *C. chrysophilum* causes anthracnose in Asia, especially in China, on oil-tea trees (Chen et al., 2022; Castillo-Cabrera et al., 2023). We cannot exclude the possibility that the identified and reported *Colletotrichum* species in Asian countries, especially from oil-tea anthracnose, can be reclassified by increasing the used loci sequences. Revaluating the classification of the reported *Colletotrichum* species causing anthracnose will be urgently required in a near future.

High sensitivity is a crucial feature of *C. fructicola* molecular markers used for practical applications. TaqMan real-time PCR targeting the ApMat gene has a detection threshold of 1 ng *C. fructicola* genomic DNA (He et al., 2023). A species-specific hydrolysis probe real-time PCR assay that combines CHLAD and FRLAD primer-probe sets improves the detection sensitivity to 5 pg fugnal genomic DNA in apple (McHenry and Aćimović, 2024). The detection threshold of *C. fructicola* using a two-step nested PCR assay in strawberry reaches 1 pg genomic DNA (Chung et al., 2022). Our study achieved a comparable detection limit of 0.63 pg DNA with qPCR assays using v013293 and v004178 primer pairs, and the v012077 primed qPCR detected as little as 0.063 pg *C. fructicola* genomic DNA. The high sensitivity of the *C. fructicola* molecular markers developed in this work enables us to detect the presence of *C. fructicola* fungal pathogen with a single qPCR assay.

Our results showed that end-point PCR and qPCR with primer pairs targeting three genes v012077, v013293, and v004178 are able to generate specific amplicons from *C. fructicola*-oil-tea *Camellia* genomic DNA mixture (Figure 4, Table 1). Therefore, these findings rule out the possibility that DNA extracted from the leaves of oil-tea *Camellia* may interfere with the specificity of *C. fructicola*-specific primers for detecting the pathogen. Previously, we identified a *TUB* primer pair that can be used in various oil-tea *Camellia* species to normalize qPCR results for the relative quantification of fungal biomass *in planta* (Cao et al., 2023). These three *C. fructicola*-specific primer sets, in combination with the oil-tea *Camellia* TUB primer, can be potentially applied for practical quantitative evaluation of *C. fructicola* in oil-tea *Camellia* orchards regardless of the cultivated tree cultivar/species. As *C. fructicola* endangers a broad list of economically important host plants (Cannon et al., 2012; Fu et al., 2019; Li et al., 2021, 2022; Martin et al., 2021; Evallo et al., 2022; Zhao et al., 2022), the application of these *C. fructicola*-specific primers in the detection of the pathogen from other vulnerable plants for quarantine can be included in future works. Moreover, these *C. fructicola*-specific primers, in combination with suitable plant report genes, have the potential to be used as an efficient tool to study the dynamics of *C. fructicola* growth in the cognate host, which is essential for *in vivo* fundamental studies of *C. fructicola*-host plant interactions.

In conclusion, we developed three *C. fructicola*-specific molecular markers that can accurately distinguish *C. fructicola* from other oil-tea *Camellia* anthracnose isolated fungal pathogens, including from these closely related fungal species belonging to *C. gloeosporioides* complex. The *C. fructicola*-specific molecular markers based on end-point PCR and qPCR assays can be used for rapid and accurate detection and identification of this fungal pathogen from inoculated leaves of oil-tea *Camellia*, thus providing valuable tools for *Camellia* anthracnose monitoring caused by *C. fructicola*. We are confident in the sanitary applications of these three *C. fructicola*-specific molecular markers in oil-tea *Camellia* nurseries and orchards.

## Data Availability

The datasets presented in this study can be found in online repositories. The names of the repository/repositories and accession number(s) can be found in the article/Supplementary material.

## Supplementary material

Supplementary Figure S1 Prediction of introns in selected *C. fructicola* unique genes.

Supplementary Table S1 Primers targeting selected genes for *C. fructicola* identification.
