# Supplementary figures and images for: Identification of specific genes as molecular markers for rapid and accurate detection of oil-tea Camellia anthracnose pathogen Colletotrichum fructicola in China
Source: Front Microbiol. 2024 Aug 26;15:1442922. doi: 10.3389/fmicb.2024.1442922 (PMC11381303; doi:10.3389/fmicb.2024.1442922)

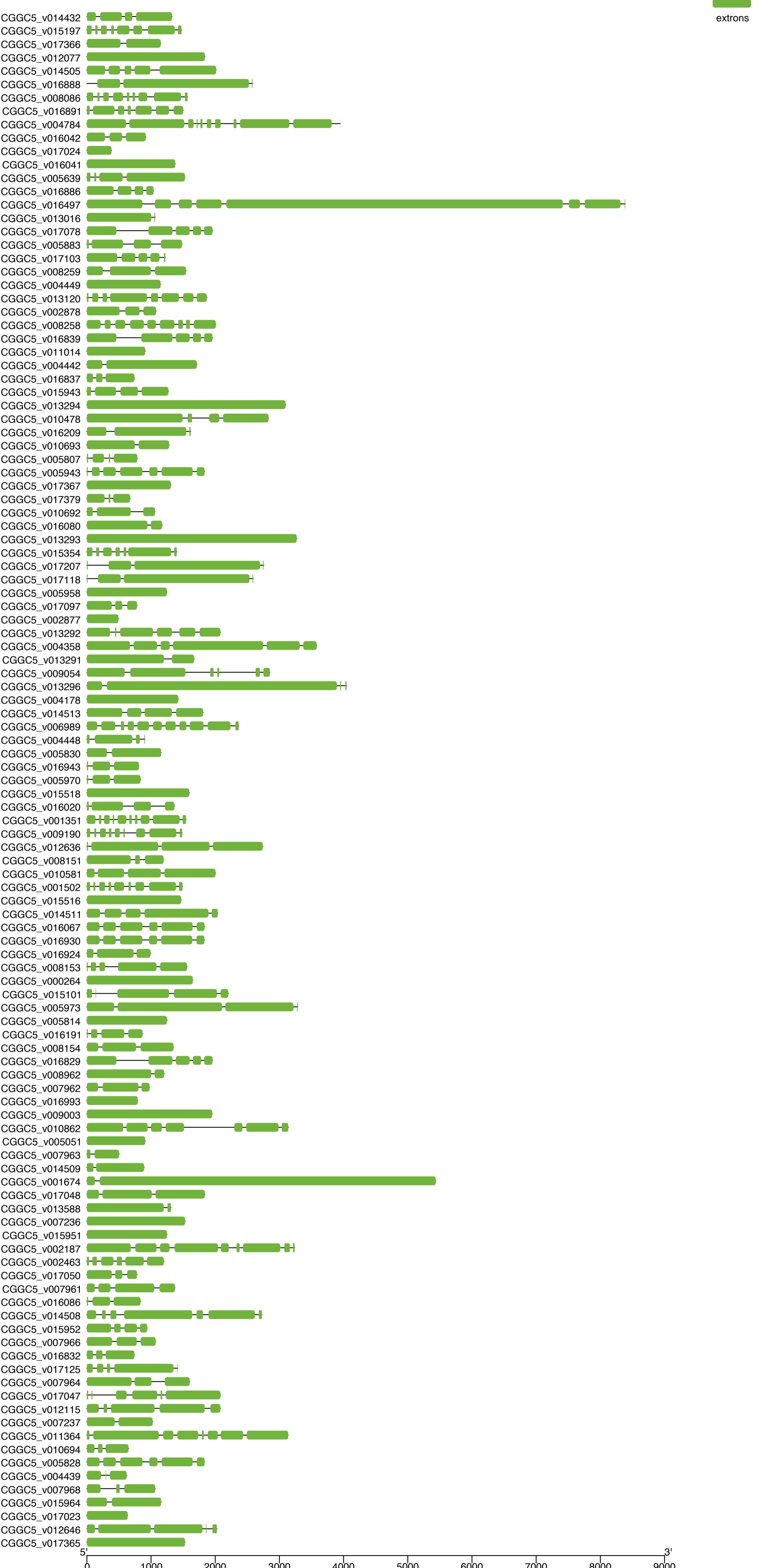

Supplement: Supplementary Figure S1 — Prediction of introns in selected C. fructicola unique genes. [file Data_Sheet_1.PDF]
