# Supplementary material for: Identification of specific genes as molecular markers for rapid and accurate detection of oil-tea Camellia anthracnose pathogen Colletotrichum fructicola in China
Source: Front Microbiol. 2024 Aug 26;15:1442922. doi: 10.3389/fmicb.2024.1442922 (PMC11381303; doi:10.3389/fmicb.2024.1442922)
Supplement: Supplementary Table S1 — Primers targeting selected genes for C. fructicola identification. [file Table_1.docx]

**Supplementary Table S1.**

Primers targeting selected genes for *C. fructicola* identification.

| **Primers** | **Sequence (5’-3’)** |
| --- | --- |
| TUB-F | CCATGCCTTGGATCACATTT |
| TUB-R | TGGGGCCATTAATGTAGACG |
| v012077-F | GGCGTCCCATTCGATCTTCA |
| v012077-R | AGCAGCAAACAAAGGGGACT |
| v017024-F | TTGCGGAAGCGAATTTGGTG |
| v017024-R | GTAGGATGCATTCGGCAGGA |
| v016041-F | ACAGAGAGGCAAGTTGCACA |
| v016041-R | TAAACGGAGCGAAGAGCGGA |
| v004449-F | ATCGCCTAATTCCTCAGCCG |
| v004449-R | CGCCTTCGGTATGCTCTAGG |
| v011014-F | TGCTCCCTTACTGTTGAGCG |
| v011014-R | CCTTCGGGGATGCAAAGGAT |
| v013294-F | CGCGCATACTTACGGCAAAA |
| v013294-R | GGGATGAGATGGCTCGGAAG |
| v017367-F | TTTTCTGAATCGCCGACGGA |
| v017367-R | ATGGTGGACTGCTTCAGGTG |
| v013293-F | CTGATCCTCACGTCGCTCTC |
| v013293-R | GCACAGGTTTCCGTGGGATA |
| v005958-F | TGATGCGTGCCATGATAGACA |
| v005958-R | TGAATCCTGCAGAAATCCGACT |
| v002877-F | TACCGGCCTTGGAAAGCAAT |
| v002877-R | CCTTCTTCTGTGAGGCCAGG |
| v004178-F | CTTCCCCGTATCGAGCTTCC |
| v004178-R | TTGAGCTCGGCGAGTTTCTT |
| v015518-F | GGCTTTTGTGCGTGGTCAAT |
| v015518-R | TCGCCGGGTTATTTGAGCTT |
| v015516-F | TCAGCCATTGTGTCGCTTCT |
| v015516-R | GTCTAACCAGGGGACTTGCC |
| v000264-F | ATACGAGGACTGGGACTGCT |
| v000264-R | GGCTTTCGACCCTTTCCGTA |
| v005814-F | GCAGTACCGTGTGGATGTGA |
| v005814-R | GAGCCGTTGCTTCCTTGTTG |
| v016993-F | GACGACAAGCTGAGGGTCAA |
| v016993-R | CGGGAATTCACCAGACCGAA |
| v009003-F | GTGCTGATCCGACTGACCAA |
| v009003-R | ACGTACGGAAGTGTTCCACC |
| v005051-F | GAACCTGCCCAAGGGATCAA |
| v005051-R | ATGCCGTCAAACGACTCACT |
| v007236-F | ACCCACCTTACGCAGATTGG |
| v007236-R | TCCCACGTTGAACATCCCAG |
| v015951-F | TCCACCGTCTCATCAGCCTA |
| v015951-R | AGCCGCACGAGCTTATCTAC |
| v017023-F | CTCTCTCCAGAGCCCTTTGC |
| v017023-R | CAGTAGCGGCAAACATGACG |
| v017365-F | TTCCATGGAGAACAACCCCG |
| v017365-R | ATGATGCGGTCGTAGAGCTG |
